# Paediatric appendicitis: international study of management in the COVID-19 pandemic

**DOI:** 10.1093/bjs/znac239

**Published:** 2022-07-19

**Authors:** Paul van Amstel, Ali El Ghazzaoui, Nigel J Hall, Tomas Wester, Francesco Morini, Johanna H van der Lee, Georg Singer, Agostino Pierro, Augusto Zani, Ramon R Gorter, the CONNECT collaborative study group, Martin L Metzelder, Sophie Langer, Ashrarur R Mitul, Sabbir Karim, Nazmul Islam, Anna Poupalou, Marc Miserez, Edward Willems, Erika V P Ortolan, Pedro Luiz Toledo de Arruda, Lourenção, Mark Bremholm Ellebæk, Susanna Petersen, Janne Suominen, Mikko Pakarinen, Françoise Schmitt, Arnaud Bonnard, Louise Montalva, Garance Martin, Antonella Nahom di eroli, Zaki Assi, Alessio Pini-Prato, Ilaria Falconi, Daniele Alberti, Giovanni Boroni, Beatrice Montanaro, Antonino Morabito, Andrea Zulli, Riccardo Coletta, Carmelo Romeo, Enrica Antonelli, Francesca Nascimben, Piergiorgio Gamba, Alberto Sgro, Alessandro Raffaele, Maria Ruffoli, Ivan Aloi, Simone Frediani, Marco Gasparella, Paola Midrio, Mohit Kakar, Shireen A Nah, Yohesuwary Gunarasa, Toni Risteski, Vesna Naunova Cvetanoska, Lazo Jovcheski, Kjetil Juul Stensrud, Henrik Røkkum, Pål Aksel Næss, Aline Vaz-Silva, Joana Patena Forte, Pedro F Morais, Joana Queirós Pereira, Sanja Sindjic Antunovic, Marija Lukac, Marion Arnold, Martina Ichino, Andrew Victor Bernstein, Hettie le Roux, Leopoldo Martinez, Carlos Delgado-Miguel, Paolo Bragagnini Rodriguez, Paula Salcedo Arroyo, Yurema Gonzalez Ruiz, Anna Svenningsson, Joep P M Derikx, Roel Bakx, Rene M H Wijnen, Claudia M G Keyzer, Gerda W Zijp, E A Huurman, Wim van Gemert, Olivier Theeuws, Ivo de VBlaauw, Sanne M B I Botden, Maja Joosten, Evert-Jan Boerma, Donald Schweitzer, Osman Uzunlu, Ingo Jester, Ben Martin, Hetal N Patel, Dina Fouad, Christine Lam, Clint D Cappiello, Carla Lopez, Veronica Natale, Emily Lee

**Affiliations:** Department of Paediatric Surgery, Emma Children’s Hospital, Amsterdam UMC, University of Amsterdam and Vrije Universiteit Amsterdam, Amsterdam, the Netherlands; Division of General and Thoracic Surgery, Hospital for Sick Children, Toronto, Ontario, Canada; University Surgery Unit, Faculty of Medicine, University of Southampton, Southampton, UK; Department of Paediatric Surgery, Astrid Lindgren Children’s Hospital, Karolinska University Hospital, Stockholm, Sweden; Department of Women’s and Children’s Health, Karolinska Institutet, Stockholm, Sweden; Neonatal Surgery Unit, Medical and Surgical Department of the Fetus, Newborn, and Infant, Bambino Gesù Children’s Hospital, IRCCS, Rome, Italy; Pediatric Clinical Research Office, Amsterdam UMC, University of Amsterdam & Vrije Universiteit Amsterdam, Amsterdam, the Netherlands; Knowledge Institute of the Dutch Association of Medical Specialists, Utrecht, the Netherlands; Department of Paediatric and Adolescent Surgery, Medical University of Graz, Graz, Austria; Division of General and Thoracic Surgery, Hospital for Sick Children, Toronto, Ontario, Canada; Department of Surgery, University of Toronto, Toronto, Ontario, Canada; Division of General and Thoracic Surgery, Hospital for Sick Children, Toronto, Ontario, Canada; Department of Surgery, University of Toronto, Toronto, Ontario, Canada; Department of Paediatric Surgery, Emma Children’s Hospital, Amsterdam UMC, University of Amsterdam and Vrije Universiteit Amsterdam, Amsterdam, the Netherlands

## Introduction

The COVID-19 pandemic had a huge impact on healthcare systems worldwide, forcing policymakers to reorganize hospital resources to prioritize COVID care. Recommendations were made by surgical societies to postpone elective surgery and apply non-operative alternatives if available for surgical diseases^1–3^. The aim of this multicentre international study was to investigate the impact of the COVID-19 pandemic on paediatric appendicitis, specifically the proportion of children with complex appendicitis, alterations in the diagnostic work-up and treatment strategies, and its outcomes.

## Methods

An international retrospective study was conducted at 40 hospitals from 23 countries (*Appendix S1*). The study was overseen by an international study steering group (RG/AZ/AP/NH/TW/FM/AG/PA) that developed the study protocol. This study was endorsed by the European Paediatric Surgeons’ Association (EUPSA), which assisted in the recruitment of participating hospitals through the EUPSA Network Office. Principal investigators of participating sites obtained local ethical approval in accordance with local requirements. The study was reported according to the STROBE guidelines^4^.

Patients (aged less than 18 years) treated for acute appendicitis between January 2019 and December 2020 were screened for eligibility. Those who had non-operative treatment without an imaging-confirmed diagnosis of acute appendicitis were excluded. Diagnosis of acute appendicitis was defined by intraoperative and histopathological confirmation of appendicitis, and, in the event of non-operative treatment (*Appendix S2*), based on clinical, biochemical, and radiological criteria. Local investigators were asked to define the start of the COVID-19 pandemic at their institution based on the start of the interval during which regular healthcare was affected by the pandemic. The COVID group included patients treated between the start of the COVID period and 31 December 2020. The control group consisted of patients treated during the corresponding interval in 2019. To understand healthcare protocols and management strategies for acute appendicitis before and during the pandemic at each centre, all participating sites were asked to complete a survey that was sent on 23 March 2021 (*Appendix S3*).

Variables of interest and their definitions were agreed by the study steering group based on the globally supported core outcome set for studies reporting the treatment of acute simple appendicitis in children^5–8^. Primary outcomes were the proportions of children treated for complex appendicitis, children who underwent imaging procedures for confirmation of appendicitis, children treated using non-surgical treatment strategies, and complications directly related to treatment. Secondary outcomes and definitions are shown in *Appendix S2*.

Comparative analyses were undertaken by calculating differences in proportions and 95 per cent confidence intervals. Subgroup analyses based on time interval of presentation, age, and region were performed for appendicitis severity and complications. For all subgroup analyses, Bonferroni correction was applied to adjust for multiple testing. Statistical analyses were carried out using SPSS^®^ version 26 (IBM, Armonk, NY, USA).

## Results

Between January 2019 and December 2020, some 10 655 children were treated for acute appendicitis, of whom 2062 were excluded for the reasons outlined in *Appendix S4*. Therefore, 8593 patients were included, 4113 in the COVID group and 4480 in the control group. Baseline characteristics were similar in the two groups (*Appendix S5*).

The survey showed that, in the majority of participating centres, non-operative treatment and same-day discharge were not standard care during the pandemic, and that there was no change in referral pathways or shift of patients with complex disease to the participating centres (*Appendix S1*).

### Appendicitis severity

In the COVID group, 47.7 per cent of patients were treated for complex appendicitis *versus* 45.0 per cent in the control group (difference 2.7 (95 per cent c.i. 0.6 to 4.8) per cent; *P* = 0.014) (*Table 1*). This increased proportion of complex appendicitis was apparent only during the first 3 months of the pandemic (difference 5.6 (1.8 to 9.3) per cent; *P* = 0.003, adjusted *P* = 0.007) and was predominantly caused by an absolute decrease in patients with simple appendicitis. The subgroup analysis based on region showed that the proportion of patients treated for complex appendicitis increased by 3.5 (1.2 to 5.8) per cent in Europe (*P* = 0.004, adjusted *P* = 0.020). No differences were found for other continents (*Table 2*).

**Table 1:** Diagnostic work-up, treatment, and outcomes

	COVID-19 group (*n* = 4113)	Control group (*n* = 4480)	Difference in proportions (%)*	*P*‡
**Severity of appendicitis**				
Simple	1973 (48.0)	2229 (49.8)	2.7 (0.6, 4.8 )	0.014
Complex	1962 (47.7)	2017 (45.0)		
Non-inflamed	168 (4.1)	220 (4.9)		
Missing	10 (0.2)	14 (0.3)		
**Patients screened for COVID-19**				
Yes	3095 (75.2)	–		
No	997 (24.2)	–		
Missing	21 (0.5)	–		
Test results				
Positive	71 (1.7)	–		
Negative	2998 (72.9)	–		
Inconclusive/unknown	26 (0.6)	–		
**No. of patients who underwent diagnostic imaging**	3535 (86.0)	3780 (84.4)	1.6 (0.1, 3.1)	0.036
Ultrasonography	3205 (77.9)	3450 (77.0)		
MRI	9 (0.2)	5 (0.1)		
CT	88 (2.1)	66 (1.5)		
Ultrasonography + MRI	59 (1.4)	63 (1.4)		
Ultrasonography + CT	161 (3.9)	164 (3.7)		
MRI + CT	1 (< 0.1)	1 (< 0.1)		
Ultrasonography + MRI + CT	1 (< 0.1)	3 (0.1)		
Missing	11 (0.3)	28 (0.6)		
**Treatment strategy**				
Non-operative	316 (7.7)	327 (7.3)	0.4 (−0.7, 1.5)	0.499
Surgical	3797 (92.3)	4153 (92.7)	0.4 (−0.7, 1.5)	0.499
**Surgical approach**				
Laparoscopic	2748 (66.8)	2889 (64.5)	2.3 (0.3, 4.3)	0.023
Open	967 (23.5)	1191 (26.6)	3.1 (1.3, 4.9)	0.001
Laparoscopic converted to open	75 (1.8)	69 (1.5)		
Other	7 (0.2)	4 (0.1)		
**Negative appendicectomy**	168 (4.1)	220 (4.9)	0.8 (−0.8, 1.7)	0.064
Missing	10 (0.2)	14 (0.3)		
**Initial duration of hospital stay (days)†**	3 (2–6)	3 (2–6)		0.751§
Missing	2 (< 0.1)	41 (0.9)		
**Total duration of hospital stay (days)†**	4 (2–6)	4 (2–6)		0.373§
Missing	2 (< 0.1)	42 (0.9)		
**Readmission**	226 (5.5)	232 (5.2)	0.3 (−0.7, 1.3)	0.532
Missing	5 (0.1)	4 (0.1)		
**No. of patients with a complication**	478 (11.6)	496 (11.1)	0.5 (−0.8, 1.8)	0.434
Missing	4 (0.1)	8 (0.2)		
**Complication severity**				
Patients with a minor complication (CD I–II)	325 (7.9)	323 (7.2)	0.7 (−0.4, 18)	0.225
Patients with a severe complication (CD III–IV)	149 (3.6)	168 (3.7)	0.1 (−0.7, 8.9)	0.754
Death (CD V)	1 (<0.1)	1 (<0.1)	0 (−0.1, 0.1)	>0.999
Missing	8 (0.2)	12 (0.3)		
**Type of complication**				
Intra-abdominal abscess	254 (6.2)	242 (5.4)		
Surgical-site infection	110 (2.7)	102 (2.3)		
Small bowel obstruction	52 (1.3)	43 (1.0)		
**Need for reoperation**	72 (1.8)	102 (2.3)	0.5 (−0.1, 1.1)	0.084
**No. of patients with at least one outpatient visit**	1898 (46.1)	2791 (62.3)	16.2 (14.1, 18.3)	<0.001
Missing	3	6		
**No. of patients with telephone check-up**	777 (18.9)	416 (9.3)	9.6 (8.1, 11.1)	<0.001
Missing	5 (0.1)	11 (0.2)		

Values are *n* (%) unless otherwise indicated; *values in parentheses are 95 per cent confidence intervals and †values are median (i.q.r.). CD, Clavien–Dindo grade. ‡Chi-Square test, except §Mann-Whitney U test.

**Table 2:** Subgroup analyses of severity of appendicitis and complications

	COVID-19 group (*n* = 4113)	Control group (*n* = 4480)	Difference in proportions (%)*	Adjusted *P*†
**Patients treated for complex appendicitis**				
Time interval				
First 3 months	672 of 1287 (52.2)	669 of 1436 (46.6)	5.6 (1.8, 9.3)	0.007
Rest of year	1290 of 2816 (45.8)	1348 of 3030 (44.5)	1.3 (−1.3, 3.9)	0.64
Missing	10	14		
Age (years)				
< 6	357 of 501 (71.3)	399 of 590 (67.6)	3.7 (−1.8, 9.1)	0.56
6–12	1018 of 2227 (45.7)	1025 of 2354 (43.5)	2.2 (−0.7, 5.1)	0.4
> 12	587 of 1375 (42.7)	593 of 1522 (39.0)	3.7 (0.1, 7.3)	0.13
Missing	10	14		
Region				
Europe	1632 of 3406 (47.9)	1593 of 3585 (44.4)	3.5 (1.2, 5.8)	0.02
North America	136 of 334 (40.7)	154 of 355 (43.4)	2.7 (−4.7, 10.0)	>0.99
South America	22 of 47 (46.8)	59 of 102 (57.8)	11.0 (−6.0, 27.3)	0.63
Africa	86 of 117 (73.5)	108 of 157 (68.8)	4.7 (−6.3, 15.2)	>0.99
Asia	86 of 199 (43.2)	103 of 267 (38.6)	4.6 (−4.4, 13.5)	0.95
Missing	10	14		
**Subgroup analysis of patients experiencing a complication**				
Time interval				
First 3 months	176 of 1291 (13.6)	173 of 1435 (12.1)	1.5 (−1.0, 4.0)	0.48
Rest of year	302 of 2818 (10.7)	323 of 3037 (10.6)	0.1 (−1.5, 1.7)	>0.99
Missing	4	8		
Age (years)				
< 6	75 of 503 (14.9)	100 of 589 (17.0)	2.1 (−2.3, 6.4)	>0.99
6–12	246 of 2230 (11.0)	252 of 2361 (10.7)	0.3 (−1.5, 2.1)	>0.99
> 12	157 of 1376 (11.4)	144 of 1522 (9.5)	1.9 (−0.3, 4.2)	0.28
Missing	4	8		
Region				
Europe	404 of 3408 (11.9)	386 of 3592 (10.7)	1.2 (−0.2, 2.7)	0.57
North America	28 of 334 (8.4)	34 of 354 (9.6)	1.2 (−3.2, 5.5)	>0.99
South America	5 of 47 (10.6)	24 of 102 (23.5)	12.9 (−1.0, 23.8)	0.33
Africa	24 of 121 (19.8)	32 of 157 (20.4)	0.6 (−9.1, 9.9)	>0.99
Asia	17 of 199 (8.5)	20 of 267 (7.5)	1.0 (−3.9, 6.4)	>0.99
Missing	4	8		

Values are *n* (%) unless otherwise indicated; *values in parentheses are 95 per cent confidence intervals. †Chi-Square test, with Bonferroni correction.

### Diagnostic work-up and initial treatment

In the COVID group, 86.0 per cent of children underwent imaging during diagnostic work-up compared with 84.4 per cent in the control group (difference 1.6 (95 per cent c.i. 0.1 to 3.1) per cent; *P* = 0.037). During the pandemic, 7.7 per cent of patients had non-operative treatment compared with 7.3 per cent in the control group (difference 0.4 (–0.7 to 1.5) per cent; *P* = 0.495). Outcomes of non-operative treatment are recorded in *Appendix S6*. Among those treated surgically, 74.3 per cent in the COVID group and 71.2 per cent in the control group underwent laparoscopic appendicectomy (difference 3.1 (1.2 to 5.1) per cent; *P* = 0.002) (*Table 1*).

### Complications

Both the primary and subgroup analyses showed no differences in the number of patients experiencing any complication between the COVID and control groups, nor in the severity of complications. In both groups, intra-abdominal abscess was the most frequent postoperative complication (*Tables 1* and *2*).

## Discussion

This large international study found that the number of patients presenting with simple appendicitis decreased during the first months of the pandemic, resulting in a higher proportion of complex appendicitis than in the control interval. The proportion of patients who had non-operative treatment and the proportion of complications were comparable to those in the control period. These data suggest that the management and outcomes of children with acute appendicitis were relatively unaffected by the pandemic, reflecting the resilience of the participating centres.

Several small single-centre studies^9–14^ have reported contradictory results on the influence of the pandemic on the proportion of patients treated for complex appendicitis; some reported increased proportions of complex appendicitis (7–18 per cent), whereas others could not detect any difference. In the present study, the increased proportion of complex appendicitis seems to be the result of an absolute decrease in patients with simple appendicitis. A possible explanation could be the resolution of mild cases of simple appendicitis in patients who did not seek medical care and recovered spontaneously or were treated with antibiotics by general practitioners^15,16^. After the first months of the pandemic, proportions of simple and complex appendicitis were comparable to those in the control group, which was predominantly the result of an absolute increase in patients with simple appendicitis. This could be explained by the fact that the threshold for seeking medical care for mild appendicitis possibly decreased after the first few months of the pandemic, as lockdown measures were slowly lifted and COVID-19-related fear declined. These findings are in line with those of other population-based studies^17–19^ that noted an absolute decrease in both adult and paediatric patients presenting with simple appendicitis early in the pandemic.

This international multicentre study is limited by possible information and selection bias, which is inherent to retrospective selection of patients and data collection. Furthermore, the regional subgroup analysis was limited by a skewed distribution, as the majority of patients were included in Europe. Finally, the survey found no shift of patients with complex disease to participating centres, but this might still have occurred. The major strength of this study is the international collaboration and subsequent large sample size of more than 8500 patients.

## Collaborators

CONNECT collaborative study group: Martin L. Metzelder, Sophie Langer (Medical University of Vienna, Vienna, Austria)

Ashrarur R Mitul, Sabbir Karim, Nazmul Islam (Bangladesh Shishu Hospital & Institute, Dhaka, Bangladesh)

Anna Poupalou, (Université Libre de Bruxelles (ULB), HUDERF Hospital (Hôpital Universitaire des Enfants Reine Fabiola), Brussels, Belgium)

Marc Miserez, Edward Willems (University Hospital Gasthuisberg KULeuven, Leuven, Belgium)

Erika V. P. Ortolan, Pedro Luiz Toledo de Arruda

Lourenção, (Botucatu Medical School, Unesp, Botucatu, São Paulo, Brazil)

Mark Bremholm Ellebæk, Susanna Petersen (Odense University Hospital, University of Southern Denmark, Odense C, Denmark)

Janne Suominen, Mikko Pakarinen (New Children's Hospital, University of Helsinki and Helsinki University Hospital, Helsinki, Finland)

Françoise Schmitt (University Hospital of Angers, Angers, France)

Arnaud Bonnard, Louise Montalva, Garance Martin (Robert Debré Children University Hospital, APHP, Paris University, Paris, France)

Antonella Nahom di Veroli, Zaki Assi (Soroka Medical Center, Beer Sheva, Israel)

Alessio Pini-Prato, Ilaria Falconi (Umberto Bosio Center for Digestive Diseases, The Children Hospital, AO SS Antonio e Biagio e cesare Arrigo, Alessandria, Italy)

Daniele Alberti, Giovanni Boroni, Beatrice Montanaro (University of Brescia, Department of Pediatric Surgery- Children's Hospital, Brescia-Italy)

Antonino Morabito, Andrea Zulli, Riccardo Coletta (University of Florence-Meyer Children's Hospital, Florence, Italy)

Carmelo Romeo, Enrica Antonelli, Francesca Nascimben (Unit of Pediatric Surgery, University of Messina, Messina, Italy)

Piergiorgio Gamba, Alberto Sgro (University of Padua, Padova, Italy)

Alessandro Raffaele, Maria Ruffoli (Fondazione IRCCS Policlinico San Matteo, University of Pavia, Pavia, Italy)

Ivan Aloi, Simone Frediani (Bambino Gesù Children's Hospital, IRCCS, Rome, Italy)

Marco Gasparella, Paola Midrio (Ca Foncello Hospital-Treviso, University of Padova, PadovaItaly)

Mohit Kakar (Riga Stradins University & Children's Clinical University Hospital. Riga, Latvia)

Shireen A Nah, Yohesuwary Gunarasa (Faculty of Medicine, University of Malaya, Kuala Lumpur, Malaysia)

Toni Risteski, Vesna Naunova Cvetanoska, Lazo Jovcheski (Ss. Cyril and Methodius University, Skopje, R. Macedonia)

Kjetil Juul Stensrud, Henrik Røkkum, Pål Aksel Næss (Oslo University Hospital, Oslo, Norway)

Aline Vaz-Silva, Joana Patena Forte, Pedro F. Morais, Joana Queirós Pereira (Hospital Dona Estefânia, Centro Hospitalar Universitário de Lisboa Central, Lisbon, Portugal)

Sanja Sindjic Antunovic, Marija Lukac (University Children’s Hospital, Belgrade, Serbia)

Marion Arnold, Martina Ichino, Andrew Victor Bernstein, Hettie le Roux (Red Cross War Memorial Children’s Hospital/University of Cape Town, Cape Town, South Africa)

Leopoldo Martinez, Carlos Delgado-Miguel, (La Paz Children's Hospital, Madrid, Spain)

Paolo Bragagnini Rodriguez, Paula Salcedo Arroyo, Yurema Gonzalez Ruiz (University Hospital “Miguel Servet”, Zaragoza, Spain)

Anna Svenningsson (Astrid Lindgren Children’s Hospital, Karolinska University Hospital, Stockholm, Sweden)

Joep PM Derikx, Roel Bakx (Emma Children's Hospital, Amsterdam UMC, University of Amsterdam & Vrije Universiteit Amsterdam, Amsterdam, The Netherlands)

Rene MH Wijnen, Claudia MG Keyzer (Erasmus University Medical Centre -Sophia Children's Hospital, Rotterdam, The Netherlands)

Gerda W Zijp, EA Huurman (Juliana Children's Hospital/Haga-Hospital, The Hague, The Netherlands)

Wim van Gemert, Olivier Theeuws (Maastricht UMC, Maastricht, Netherlands)

Ivo de Blaauw, Sanne MBI Botden, Maja Joosten (Radboud University Medical Center, Amalia Children's Hospital, Nijmegen, The Netherlands)

Evert-Jan Boerma, Donald Schweitzer (Zuyderland Medical Centre, Heerlen&Sittard-Geleen, The Netherlands)

Osman Uzunlu (Pamukkale University School of Medicine, Denizli, Turkey)

Ingo Jester, Ben Martin, Hetal N Patel (Birmingham Children's Hospital, Birmingham, West Midlands, United Kingdom)

Dina Fouad, Christine Lam (Southampton Children’s Hospital, Southampton, United Kingdom)

Clint D. Cappiello, Carla Lopez, Veronica Natale, Emily Lee (The Johns Hopkins Hospital Bloomberg Children's Center, Baltimore, MD, United States of America)

## Funding

T.W. received a grant from the Swedish Medical Research Council. The Swedish Medical Research Council had no role in any part of the study.

## Supplementary Material

znac239_Supplementary_Data
