# Supplementary material for: Paediatric appendicitis: international study of management in the COVID-19 pandemic
Source: Br J Surg. 2022 Jul 19;109(11):1044–8. doi: 10.1093/bjs/znac239 (PMC9384519; doi:10.1093/bjs/znac239)
Supplement: znac239_Supplementary_Data [file znac239_supplementary_data.docx]

**Appendix S1. Definition of COVID period and healthcare regulations**

| **Country** | **Site** | **Number of patients** | **Start date COVID-19** | **NOT standard of care** | **Same day discharge standard of care** | **Shift of patients with acute appendicitis** |
| --- | --- | --- | --- | --- | --- | --- |
| Austria | Graz | 352 | March 2020 | No | No | No |
|  | Vienna | 224 | March 2020 | No | No | COVID+ patients were referred to another hospital |
| Bangladesh | Dhaka | 56 | March 2020 | No | No | No |
| Belgium | Brussels | 120 | March 2020 | No | No | No |
|  | Leuven | 161 | March 2020 | No | No | No |
| Brazil | Sao Paolo | 149 | March 2020 | No | No | No |
| Canada | Toronto | 511 | April 2020 | No | No | No |
| Denmark | Odense | 171 | March 2020 | No | No | No |
| Finland | Helsinki | 279 | March 2020 | No | No | No |
| France | Angers | 235 | March 2020 | No | No | No |
|  | Paris | 328 | March 2020 | No | Yes | Patients (both simple and complex appendicitis) were referred from peripheral hospitals to this hospital due to reduced OR capacity |
| Israel | Ber Sheva | 292 | April 2020 | No | No | No |
| Italy | Alessandria | 103 | March 2020 | No | Yes | No |
|  | Brescia | 289 | March 2020 | Yes | No | No |
|  | Florence | 242 | March 2020 | No | Yes | No |
|  | Messina | 150 | March 2020 | No | No | COVID+ patients were referred to another hospital |
|  | Padua | 106 | March 2020 | No | No | No |
|  | Pavia | 96 | March 2020 | No | No | Patients (both simple and complex appendicitis) were referred from peripheral hospitals to this hospital |
|  | Rome | 528 | March 2020 | No | No | No |
|  | Treviso | 146 | February 2020 | No | No | No |
| Latvia | Riga | 191 | February 2020 | Yes | No | No |
| Malaysia | Kuala Lumpur | 27 | February 2020 | No | No | No |
| North-Macedonia | Skopje | 244 | March 2020 | No | No | No |
| Norway | Oslo | 208 | March 2020 | No | No | No |
| Portugal | Lisbon | 580 | March 2020 | No | No | No |
| Serbia | Belgrade | 307 | March 2020 | No | No | Patients (both simple and complex appendicitis) were referred from peripheral hospitals to this hospital |
| South-Africa | Cape Town | 278 | March 2020 | Yes | No | All pediatric cases were referred to this hospital |
| Spain | Madrid | 554 | March 2020 | No | No | Emergency pediatric surgery was concentrated in this hospital |
|  | Zaragoza | 387 | March 2020 | No | No | No |
| Sweden | Stockholm | 297 | March 2020 | Yes | No | No |
| The Netherlands | Amsterdam | 29 | March 2020 | No, only in trial setting | No | No |
|  | Heerlen | 117 | March 2020 | No, only in trial setting | No | No |
|  | Maastricht | 45 | March 2020 | No | No | No |
|  | Nijmegen | 7 | March 2020 | No | No | No |
|  | Rotterdam | 33 | March 2020 | No | No | No |
|  | The Hague | 145 | March 2020 | No, only in trial setting | No | No |
| Turkey | Denizli | 91 | March 2020 | Yes | No | No |
| United Kingdom | Birmingham | 196 | April 2020 | No | No | No |
|  | Southampton | 141 | April 2020 | No, surgeon discretion | No | No |
| United States | Baltimore | 178 | April 2020 | Yes | Yes | COVID+ cases were transferred to this hospital |

**Non-operative treatment protocols:**

Brescia, Italy: Intravenous (IV) ceftriaxone for 7 days in case of simple appendicitis, ceftriaxone + metronidazole IV for complicated appendicitis.

Riga, Latvia: IV ampicillin + metronidazole for 72 hours followed by 7 days oral augmentin to complete a course of 10 days. Discharge criteria: Clinical improvement, decreased c-reactive protein, and no deterioration on repeat ultrasound.

Cape Town, South-Africa: IV augmentin for 24-48 hours followed by oral augmentin + amoxicillin to complete a course of 10 days. Discharge criteria: Clinical improvement and after confirmation that caregivers have clear understanding of symptoms to prompt early return to hospital as well as easy access to transport back to the hospital.

Stockholm, Sweden: IV piperacillin/tazobactam for 24 hours followed by oral ciprofloxacin + metronidazole to complete a course of 10 days. Discharge criteria: Clinical improvement after 24 to 48 hours.

All centers, The Netherlands: IV augmentin + gentamicin for 48 hours followed by 5 days oral augmentin. Discharge criteria: Clinical improvement, decreased leukocytes and c-reactive protein and no deterioration on repeat ultrasound.

Denizli, Turkey: IV ampicillin/sulbactam + gentamicin + clindamycin for 5 days followed by oral augmentin + metronidazole to complete a course of 10 days.

Southampton, United Kingdom: No written protocol, but a minimum of 24 hours of IV antibiotics.

Baltimore, United States: IV ceftriaxone + metronidazole for at least 24 hours followed by oral Levaquin + metronidazole to complete a course of 10-14 days. Discharge criteria: toleration of oral diet, afebrile, pain improved. In case of complicated appendicitis interval appendectomy was planned 6 to 8 weeks after discharge.

**Appendix S2. Outcomes and definitions**

**Secondary outcomes**

Children developing complex appendicitis after non-operative treatment (NOT): The proportion of children that were initially treated non-operatively for simple appendicitis and subsequently developed complex appendicitis (either during initial admission or due to recurrence of disease) within 30 days after initial treatment.

Children with recurrent appendicitis after NOT: Recurrent appendicitis was defined as histopathological confirmed acute appendicitis within 30 days after initial NOT.

Hospital readmissions: The number of patients that were readmitted for a complication related to appendicitis treatment within 30 days after initial treatment.

Need for reoperation: The number of patients that underwent a second surgical procedure in the same site for the same indication within 30 days after primary appendectomy.

Length of hospital stay: The total duration of hospital stay during the first admission (initial length of hospital stay) combined with admission due to complications related to appendectomy or appendicitis.

Number of outpatient visits: The number of regular visits and telephone check-ups related to appendicitis treatment.

**Other definitions**

Non-operative treatment (NOT): Non-operative treatment of simple appendicitis consisted of a minimum of 24 hours intravenous antibiotics with or without a subsequent course of oral antibiotics, according to local protocol. Non-operative treatment of complex appendicitis consisted of intravenous antibiotics with or without percutaneous drainage (in case of appendiceal abscess).

Successful NOT: Successful NOT was defined as no need for appendectomy during the 30-day follow-up period of the study.

Simple appendicitis: Simple appendicitis was defined as the macroscopic appearance of an increased diameter of the appendix and microscopic transmural inflammation, ulceration, or thrombosis, without signs of necrosis or perforation.^6^

Complex appendicitis: Complex appendicitis was defined as appendicitis with macroscopic or microscopic signs of transmural inflammation with necrosis (gangrenous appendicitis) or perforation, or appendicitis with abscess or mass.^6^

Appendiceal perforation: Perforation was defined as a visible hole in the appendix or a free fecalith in the abdominal cavity.^7^

Intra-abdominal abscess (IAA): IAA was defined as a radiologically confirmed accumulation of purulent fluid in a walled-off space within the abdominal cavity.

(Adhesive) bowel obstruction: The diagnosis of (adhesive) bowel obstruction was based on clinical signs and symptoms such as history of constipation, nausea, vomiting, and distended abdomen.

Surgical site infection (SSI): Superficial and deep SSI were defined according to the CDC criteria.^8^

**Appendix S3. Survey: Changes in healthcare protocols and treatment of appendicitis**

1. Please indicate the start date of the COVID-19 pandemic in your region (based on the start of the time period in which healthcare in your hospital was affected by the pandemic)

2. Did you experience different peaks in the number of patients treated for COVID-19 in your hospital? If so, please define the start and end dates of these COVID-19 peaks.

3. Please indicate the percentage of reduced operation room capacity in your hospital during the COVID-19 pandemic

4. Did your hospital change any surgical healthcare protocols regarding the treatment of acute appendicitis during the COVID-19 pandemic?

5. Was non-operative treatment preferred for children with simple appendicitis according your adjusted protocol?

6. Was same day discharge preferred for children with simple appendicitis according to your adjusted protocol?

7. Did the changes in surgical protocols and/or reduction of operation room capacity affect the treatment of children with acute appendicitis in any other way?

8. Were any healthcare protocols implemented in your country that ordered a shift in appendicitis treatment from tertiary (academic) hospitals to peripheral hospitals or vice versa?

9. If so, did this shift of patients affect treatment of children with appendicitis in your hospital? Did your hospital treat more or less children with acute appendicitis due to the adjusted healthcare protocols?

10. Do you have any additional comments regarding changes in your healthcare protocols and management strategies for acute appendicitis during the COVID-19 pandemic?

**Appendix S4. Patient flowchart**


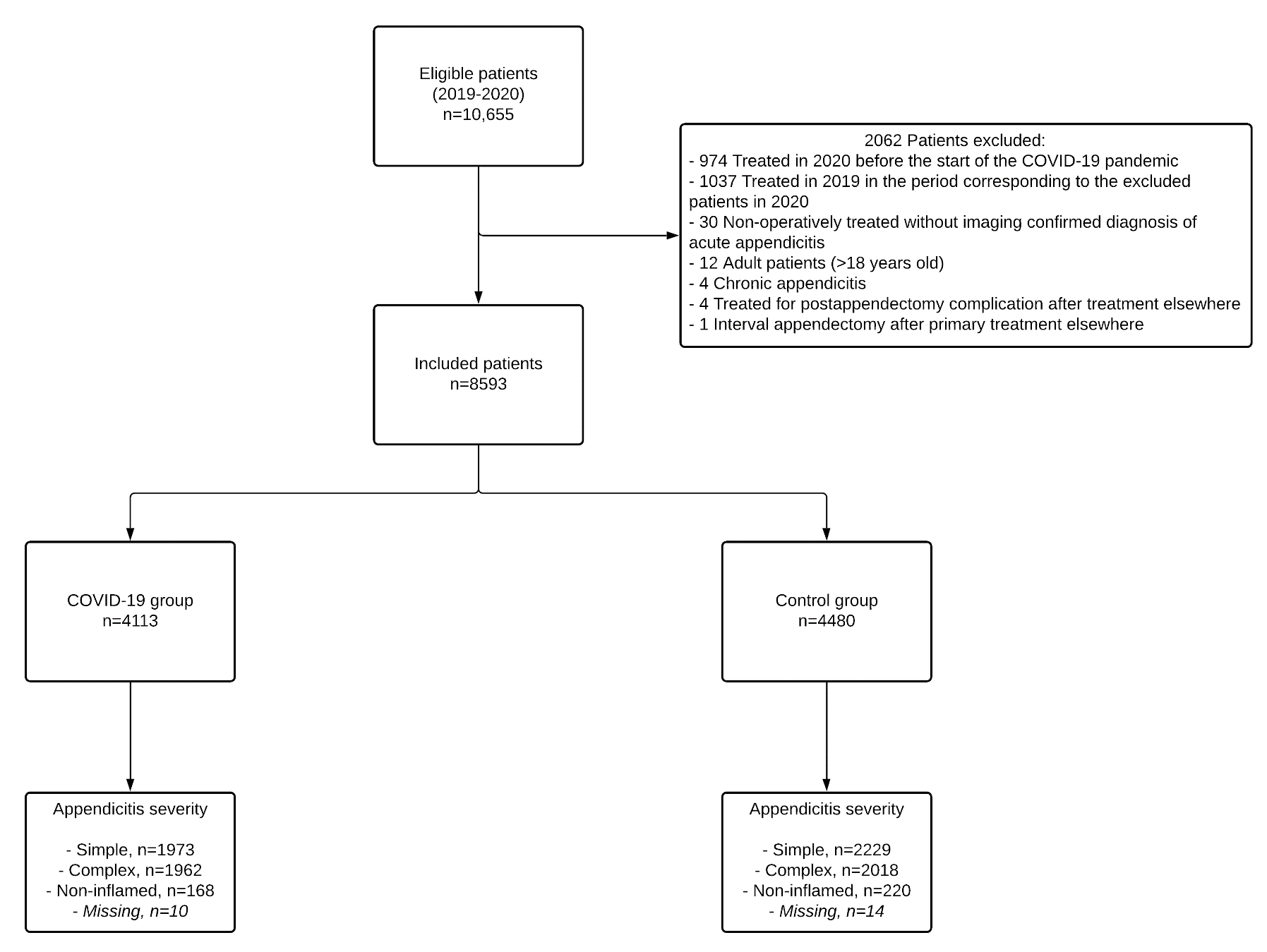


**Appendix S5. Baseline characteristics**

| **Characteristics** | **COVID-19 group (n= 4113)** | **Control group (n= 4480)** | ***p*-value** |
| --- | --- | --- | --- |
| Age, y* | 9.8 ± 3.6 | 9.9 ± 3.7 | 0.55 |
| Male sex | 2558 (62.2%) | 2689 (60.0%) | 0.04 |
| Days abdominal pain^  *Missing values* | 1 (1-2)  *36* | 1 (1-2)  *62* | 0.41 |
| Temperature at presentation*  *Missing values* | 37.2 ± 0.9  *138* | 37.2 ± 0.9  *151* | 0.09 |
| Leukocytes*  *Missing values* | 15.1 ± 5.5  *136* | 15.1 ± 5.6  *184* | 0.61 |
| CRP^  *Missing values* | 24.0 (5.0-73.0)  *160* | 20.0 (3.6-67.9)  *231* | 0.001 |
| Severity of appendicitis   - Simple - Complex - Non-inflamed   *Missing values* | 1973 (48.0%)  1962 (47.7%)  168 (4.1%)  *10 (0.2%)* | 2229 (49.8%)  2017 (45.0%)  220 (4.9%)  *14 (0.3%)* | 0.02 |
| Patients screened for COVID-19   - Yes - No   *Missing values*  Test results   - Test positive - Test negative   Inconclusive/unknown | 3095 (75.2%)  997 (24.2%)  *21 (0.5%)*  71 (1.7%)  2998 (72.9%)  26 (0.6%) | -  -  *-*  -  -  - | - |

Data displayed as count (percentage of total group count)

*Data displayed as mean ± standard deviation

^Data displayed as median (interquartile range

**Appendix S6a. Outcomes of NOT for simple appendicitis**

| **Characteristics** | **COVID-19 group (n=211)** | **Control group (n=208)** |
| --- | --- | --- |
| Need for appendectomy within one month | 27 (12.8%) | 27 (13.0%) |
| Reason for appendectomy   - Primary failure of NOT - Recurrent appendicitis | 17 (8.1%)  10 (4.7%) | 19 (9.1%)  7* (3.4%) |
| Patients developing complex appendicitis   - Primary - Secondary | 9 (4.3%)  7 (3.3%)  2 (0.9%) | 2 (1.0%)  2 (1.0%)  0 |

* One patient with a suspicion of recurrent appendicitis had a non-inflamed appendix at histopathological examination

Data is displayed as count (percentage of total)

**Appendix S6b. Outcomes of NOT for complex appendicitis**

| **Characteristics** | **COVID-19 group (n=105)** | **Control group (n=119)** |
| --- | --- | --- |
| Indication for NOT   - Appendix mass - Appendiceal abscess - Unknown | 26 (24.8%)  63 (60.0%)  16 (15.2%) | 33 (27.7%)  70 (58.8%)  16 (13.4%) |
| Need for appendectomy within one month | 14 (13.3%) | 18 (15.1%) |
| Reason for appendectomy   - Primary failure of NOT - Recurrent appendicitis - Interval appendectomy   *Missing* | 6 (5.7%)  7 (6.7%)  0  *1 (1.0%)* | 8 (6.7%)  8 (6.7%)  2 (1.7%)  *0* |

Data is displayed as count (percentage of total for each subgroup)
